# Clinical and Engagement Results of a Nationwide Comprehensive Remote Patient Care Hypertension Program

**DOI:** 10.1016/j.jacadv.2025.101892

**Published:** 2025-07-23

**Authors:** David I. Feldman, Spencer Reynolds, Luis Valor, Sarine Babikian, Theodore Feldman, Randall Curnow, Brian D. Stein, Christopher Frost, Sowmya Viswanathan, Jeffrey Galles, Lynn T. Simon, Eve Cunningham, Marat Fudim

**Affiliations:** a Department of Medicine, Massachusetts General Hospital, Boston, Massachusetts, USA; b Cadence, New York, New York, USA; c Rush University Medical Center, Chicago, Illinois, USA; d Lifepoint Health, Franklin, Tennessee, USA; e BayCare Health System, Tampa, Florida, USA; f Hillcrest Medical Center, Tulsa, Oklahoma, USA; g Community Health Systems, Franklin, Tennessee, USA; h Department of Medicine, Duke University Medical Center, Durham, North Carolina, USA

**Keywords:** health disparities, hypertension, remote patient care, remote patient monitoring, social determinants of health

## Abstract

**Background:**

Traditional methods for treating hypertension have been ineffective. Remote patient care (RPC) can transform how we provide longitudinal care for patients with hypertension.

**Objectives:**

The purpose of this study was to determine whether an RPC hypertension program can provide a scalable solution for optimizing hypertension management, especially among rural/underserved patients.

**Methods:**

A technology-enabled RPC provider enrolled patients into an RPC hypertension program from February 2022 to June 2024. Using vitals automatically transmitted from a cellular-enabled blood pressure (BP) cuff and scheduled visits, clinicians leveraged technology-enabled clinical protocols to drive engagement and improve hypertension control. Mean reduction in BP and percentage of individuals at BP goal were evaluated using a paired *t*-test and McNemar's test, respectively.

**Results:**

A total of 23,638 patients were enrolled (mean [SD] age 73(9), 60.0% female, 56.9% rural/underserved) with a baseline BP of 140/81 mm Hg. The mean (SD) reduction in BP was 7/5 (14/8) mm Hg (*P* < 0.001) after a follow-up of 30 (24) weeks, with a greater reduction in individuals with a higher baseline BP. Clinical engagement was high (75% and 57% of patients still measuring vitals at 6 and 12 months, respectively) with 11,834,837 vitals measured, 177,620 clinical encounters and 118,792 phone calls completed, and 117,457 high-acuity clinical alerts resolved. There was a 70% relative increase in the number of patients at goal BP at follow-up (36.6% vs 21.5%; *P* < 0.001). Results among rural/underserved patients were similar.

**Conclusions:**

A nationwide RPC hypertension program can drive high patient engagement and facilitate clinical encounters at scale, resulting in reduced BP levels and more patients achieving BP goals across the United States.

Hypertension is the most common modifiable risk factor for atherosclerotic cardiovascular disease in the United States.^1^ Despite decades of clinical research and public health initiatives, hypertension management continues to be inadequate even among the Medicare population. This unmet need is evident in the persistent prevalence of uncontrolled hypertension among patients, with nearly one-quarter of the adult population affected.^2^^,^^3^ The consequences of poor blood pressure (BP) control are profound, contributing to an increased risk of cardiovascular disease, stroke, and kidney failure, which collectively result in substantial morbidity, mortality, and health care costs.^4^

The financial burden associated with hypertension is staggering. Direct medical costs attributed to hypertension exceed $130 billion annually in the United States, with additional indirect costs from lost productivity and long-term disability further exacerbating the economic impact.^5^ Traditional management approaches, including lifestyle interventions, pharmacotherapy, and regular in-office BP monitoring, have proven insufficient in addressing the scope of the problem.^6^ In addition, a significant percentage of patients remain nonadherent to prescribed treatment regimens, which results in many failing to achieve target BP levels despite ongoing therapy.^7^ These failures highlight the limitations of existing strategies, particularly in ensuring continuous and effective patient engagement and monitoring to help drive improved BP control. Unfortunately, the rates of hypertension control are even lower for individuals influenced by social determinants of health (SDOH),^8^ residential environment,^9^ race/ethnicity^10^^,^^11^ and culture/language barriers.^12^

One of the emerging approaches to addressing these gaps in hypertension management is the implementation of remote patient monitoring (RPM) technologies. RPM enables consistent, real-time tracking of patients' BP and other vital signs outside of the clinical setting, offering a more dynamic and responsive approach to care. By leveraging connected devices and data analytics, RPM has the potential to enhance patient adherence, provide early detection of hypertensive crises, and facilitate timely intervention by health care providers. This comprehensive approach of providing remote patient care (RPC) has been acknowledged by guidelines as a solution to the persistent challenges in hypertension control.^1^^,^^13^

The aim of this retrospective clinical outcomes analysis was to determine whether a collaboration between a RPC partner offering RPM and technology-enabled, guideline-directed clinical interventions, and primary care and cardiology clinicians across the United States could improve the treatment of all hypertension patients nationwide. Building on experiences from various academic medical centers that demonstrated the potential of RPM to deliver remote care to patients,^14^^,^^15^ we sought to assess how a comprehensive RPC hypertension program implemented in partnership with health systems impacts scalability, patient engagement, and retention and clinical outcomes, especially among patients from rural/underserved areas.

## Methods

### Population

The RPC hypertension program described is one of three active RPC programs delivered by Cadence, which is a health care technology company that partners with large national health systems to deliver RPC to patients with chronic disease, including hypertension, diabetes, and heart failure. The comprehensive RPC program included a multidisciplinary group of clinicians such as physicians, nurse practitioners, registered nurses, and medical assistants who leveraged RPM to deliver clinical care for patients with chronic diseases alongside health care institutions and patients' longitudinal providers (10 health systems nationwide). The RPC program is integrated with each health system's electronic health record (EHR).

A remote clinical team monitored self-measured vitals on a validated cellular-enabled, automated BP cuff and heart rate monitor provided to each patient upon enrollment into the program. Virtual clinical visits using standardized clinical protocols were also conducted via telephone on a regular basis to facilitate guideline-directed clinical interventions including symptom, vital and medication optimization. In addition, a clinical team was available 24/7 to answer patient-related questions.

Patients consented in person or virtually prior to enrollment in the RPC program. Rural and underserved areas were defined according to the Health Resources and Services Administration's Federal Office of Rural Health Policy and Federal Housing Finance Agency, respectively.

### Eligibility and enrollment

Patients eligible for the retrospective analysis of the hypertension program were Medicare enrollees who had a diagnosis of hypertension based on International Classification of Diseases-10 codes, a mean BP >130/80 mm Hg (using last 3 clinic-based vitals), and were enrolled for at least 30 days between February 2022 and June 2024.

Patients were eligible to enroll in the RPC program if they were managed by a clinician at a participating health system. Eligible patients were identified via an automated, proprietary algorithm and presented to participating clinicians. If a participating clinician chose to place an order via the EMR, the patient was contacted for an in-person or virtual enrollment. Devices were provided either in person or delivered to the patient's home address.

### Patient engagement and treatment

Following an order from the patient's participating provider, a patient was contacted by a care team member to consent to participate in the RPC program, complete device setup, confirm contact information for both the patient and caretakers, and provide a preferred method of communication. Patients were counseled on the importance of daily vitals measurement, which were monitored by a team of 24/7 nurses. Automated reminders were sent to patients via SMS to encourage daily vitals measurement and improve engagement.

Proprietary alerts were triggered based on abnormal BP or heart rate measurements and managed by the clinical team. Remote visits were scheduled based on patient acuity and included guideline-based interventions ranging from behavioral and lifestyle to pharmacological recommendations from a nurse practitioner-led team.

Medication titrations were based on the multisociety clinical guidelines and according to clinical protocols approved by partner health system leadership and clinicians.^1^ Patients were eligible for different levels of clinical support from the RPC team, which was assigned based on the longitudinal clinician order. Options range from “*full medication management*,” where the RPC team initiates any clinically indicated hypertension-related decisions for the patient based on guidelines and preapproved protocols, to “*current medications only*,” where the RPC team has the ability to titrate only medications patients are already prescribed, to “*recommend only*,” where the RPC team does not titrate medications but rather recommends clinically appropriate medication changes for consideration by the ordering provider.

Baseline and follow-up BP were determined using all vitals patients measured at home during the first 2 weeks and last 2 weeks in the program, respectively, leading up to the date of analysis in July 2024. Baseline program BP was calculated using a mean of 12 home-based measured vitals per patient during the first 2 weeks following enrollment. Patient retention was measured by the number of patients who disengaged, either due to inactivity, which is defined as not taking vitals in 30 days or disenrollment from the program.

### Data

The data set used for this aggregated analysis was derived from partner health systems and the RPC platform. Patient characteristics and comorbidities (ICD-10 codes) were obtained from the EHR while individual vitals and medication data were obtained from the RPC platform. The Institutional Review Board at the Duke University Medical Center approved the retrospective review of the data.

### Statistical analysis

Continuous variables were described using mean ± SD and were compared using 2-sided paired *t*-tests to evaluate the change from baseline to follow-up. Categorical variables were described using percentages and were compared using McNemar's test to evaluate changes from baseline. A *P* value of <0.05 was deemed statistically significant. All statistical analyses were performed in Python 3.13.2 using open-source libraries.

## Results

**Figure 1:**
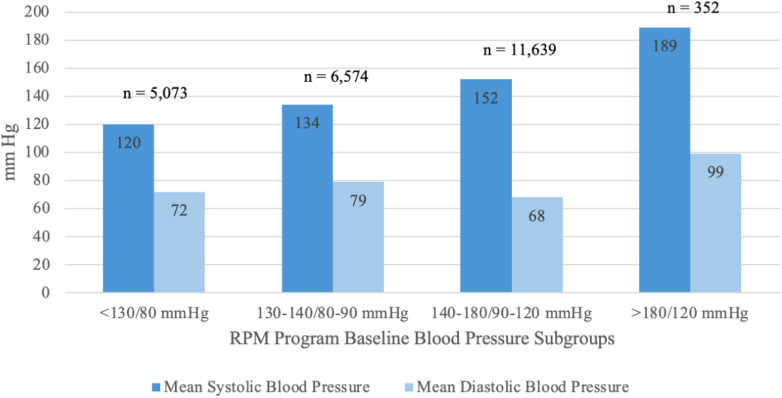
Mean RPC Program Blood Pressure by Starting Blood Pressure Subgroups RPC = remote patient care; RPM = remote patient monitoring.

A total of 23,638 patients were enrolled (mean [SD] age 73 [9] years) with a mean (SD) program baseline and clinic-based BP of 140/81 (20/11) and 140/78 (16/9) mm Hg, respectively (Central Illustration). Patients were predominantly women (60%), White (41%) or unknown race (47%), from rural or underserved geographic areas (57%), enrolled in the full medication management program (51%), and had multiple comorbidities (Table 1). Mean BP stratified by baseline acuity is highlighted in Figure 1.

**Central Illustration:**
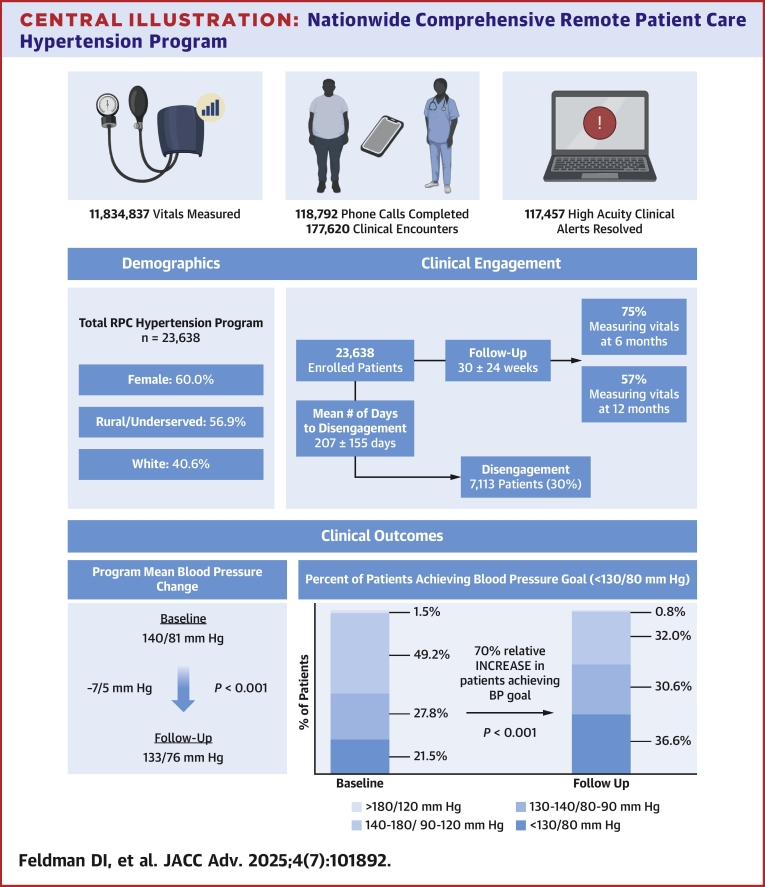
Nationwide Comprehensive Remote Patient Care Hypertension Program Abbreviations as in Figures 1 and 2.

**Table 1:** Baseline Characteristics (N = 23,638)

Age, mean (SD), y	73 (9)
Follow-up, mean (SD), wk	30 (24)
Female, n (%)	14,176 (60.0)
Race, n (%)	
Caucasian	9,598 (40.6)
Black	1,487 (6.3)
Hispanic	850 (3.6)
Other	701 (3.0)
Unknown	11,002 (46.5)
Geographic region, n (%)	
Rural/underserved	13,458 (56.9)
Nonrural/nonunderserved	10,180 (43.1)
Comorbidities, n (%)	
Coronary artery disease	4,247 (18.0)
Chronic kidney disease	5,995 (25.4)
Chronic obstructive pulmonary disease	7,875 (33.3)
Depression	1,830 (7.7)
Hyperlipidemia	18,577 (78.6)
Overweight/Obesity	7,884 (33.4)
Smoking	2,245 (9.5)
Type 2 diabetes	1,082 (4.6)
Program baseline blood pressure, mean (SD), mm Hg	140/81 (20/11)
<130/80 mm Hg	120/72 (7/5)
130-140/80-90 mm Hg	134/79 (5/6)
140-180/90-120 mm Hg	152/86 (10/8)
>180/120 mm Hg	189/99 (9/11)
In-clinic blood pressure, mean (SD), mm Hg	140/78 (16/9)
<130/80 mm Hg	132/75 (15/8)
130-140/80-90 mm Hg	137/78 (15/9)
140-180/90-120 mm Hg	144/79 (15/9)
>180/120 mm Hg	157/83 (19/11)
Baseline blood pressure acuity subgroups, n (%)	
<130/80 mm Hg	5,073 (21.5)
130-140/80-90 mm Hg	6,574 (27.8)
140-180/90-120 mm Hg	11,639 (49.2)
>180/120 mm Hg	352 (1.5)
Program type, n (%)	
Full medication management	12,025 (50.9)
Current medications only	5,126 (21.7)
Recommend only	6,333 (26.8)

### Program engagement

Clinical engagement was high with 11,834,837 vitals measured, 177,620 clinical encounters and 118,792 phone calls completed, and 117,457 high-acuity clinical alerts resolved. Individuals with baseline BP 140 to 180/90 to 120 mm Hg made up most of the absolute number of clinical engagement parameters, however individuals with a baseline BP >180/120 mm Hg had the highest per patient clinical engagement and utilization rate excluding total vitals measured (Table 2).

**Table 2:** Clinical Engagement

	Total Population (N = 23,638)	Baseline BP <130/80 mm Hg (n = 5,073)	BP 130-140/80-90 mm Hg (n = 6,574)	BP 140-180/90-120 mm Hg (n = 11,639)	Baseline BP >180/120 mm Hg (n = 352)
Vitals, total	11,834,837	2,451,080	3,274,484	5,967,795	141,478
# of vitals per patient, mean (SD)	501 (531)	483 (531)	498 (523)	513 (535)	402 (533)
# of vitals per patient, median (IQR)	317 (160-644)	301 (153-614)	318 (162-629)	326 (165-672)	219 (76-531)
Clinical encounters, total	177,620	30,504	36,643	101,734	8,739
# of clinical encounters per patient, mean (SD)	7.5 (11)	6.0 (9)	5.6 (9)	8.7 (11)	24.8 (24)
# of clinical encounters per patient, median (IQR)	4 (2-9)	3 (2-7)	3 (2-6)	5 (3-11)	18 (9-33)
Total phone calls, total	118,792	23,631	27,871	63,369	3,921
# of phone calls per patient, mean (SD)	5.0 (6)	4.7 (5)	4.2 (5)	5.4 (6)	11.1 (15)
# of phone calls per patient, median (IQR)	3 (1-7)	3 (2-6)	3 (1-6)	4 (1-7)	6 (0-17)
High-acuity clinical alerts, total	117,457	16,516	16,385	74,215	10,341
# of high-acuity clinical alerts per patient, mean (SD)	5.0 (13)	3.3 (10)	2.5 (10)	6.4 (14)	29.4 (32)
# of high-acuity clinical alerts per patient, median (IQR)	1 (0-4)	1 (0-3)	0 (0-2)	2 (0-7)	18 (8-40)

Most high-acuity alerts were related to high BP or low mean arterial pressure (MAP); the most common high-acuity alert that occurred was a high systolic BP (SBP) (n = 66,916), followed by low MAP (n = 19,141) and high diastolic BP (DBP) (n = 5,737). The majority of high SBP and DBP alerts were triggered by patients in the 140 to 180/90 to 120 mm Hg subgroup (SBP: n = 51,785; DBP: n = 4,181); however, the highest number of alerts per patient was among those with a baseline BP >180/120 mm Hg (SBP: n = 9,668; DBP: n = 683). For low MAP alerts, the majority were triggered by individuals with a baseline BP <130/80 mm Hg (n = 9,061).

Seventy-five and fifty-seven percent of individuals were still measuring vitals at 6 and 12 months, respectively. At 6 months, individuals with a baseline BP 130 to 140/80 to 90 mm Hg (77%) were most likely to still be transmitting vitals followed by <130/80 mm Hg (76%), 140 to 180/90 to 120 mm Hg (74%), and >180/120 mm Hg (57%). A similar trend was observed at 12 months, with 60% of individuals with a baseline BP 130 to 140/80 to 90 mm Hg transmitting vitals followed by < 130/80 mm Hg (59%), 140 to 180/90 to 120 mm Hg (55%), and >180/120 mm Hg (39%). Rural/underserved patients were also more likely compared to nonrural/nonunderserved patients to be measuring vitals at 6 and 12 months, respectively (6 months: 76% vs 74%; 12 months: 59% vs 55%).

### Blood pressure management

**Figure 2:**
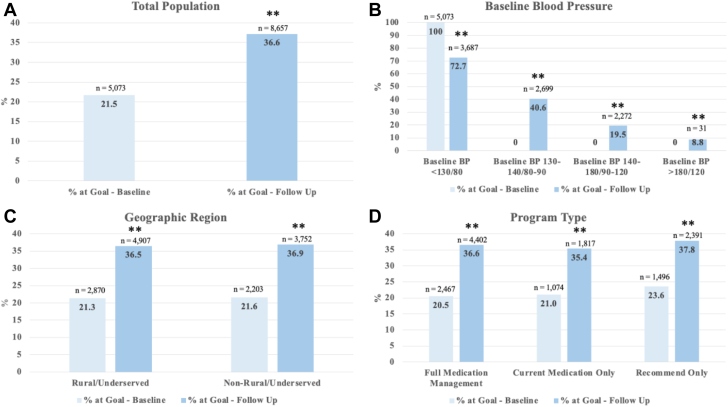
Changes in the Percentage of Patients at Goal (A) Percentage of all patients at goal blood pressure (<130/80 mm Hg) at baseline and follow-up; (B) percentage of patients at goal blood pressure at baseline and follow-up according to baseline blood pressure; (C) percentage of patients at goal blood pressure at baseline and follow-up according to geographic region; (D) percentage of patients at goal blood pressure at baseline and follow-up according to program type. Statistical significance was determined using McNemar's test. Statistical significance is reported as follows: ∗∗*P* < 0.01. BP = blood pressure.

**Figure 3:**
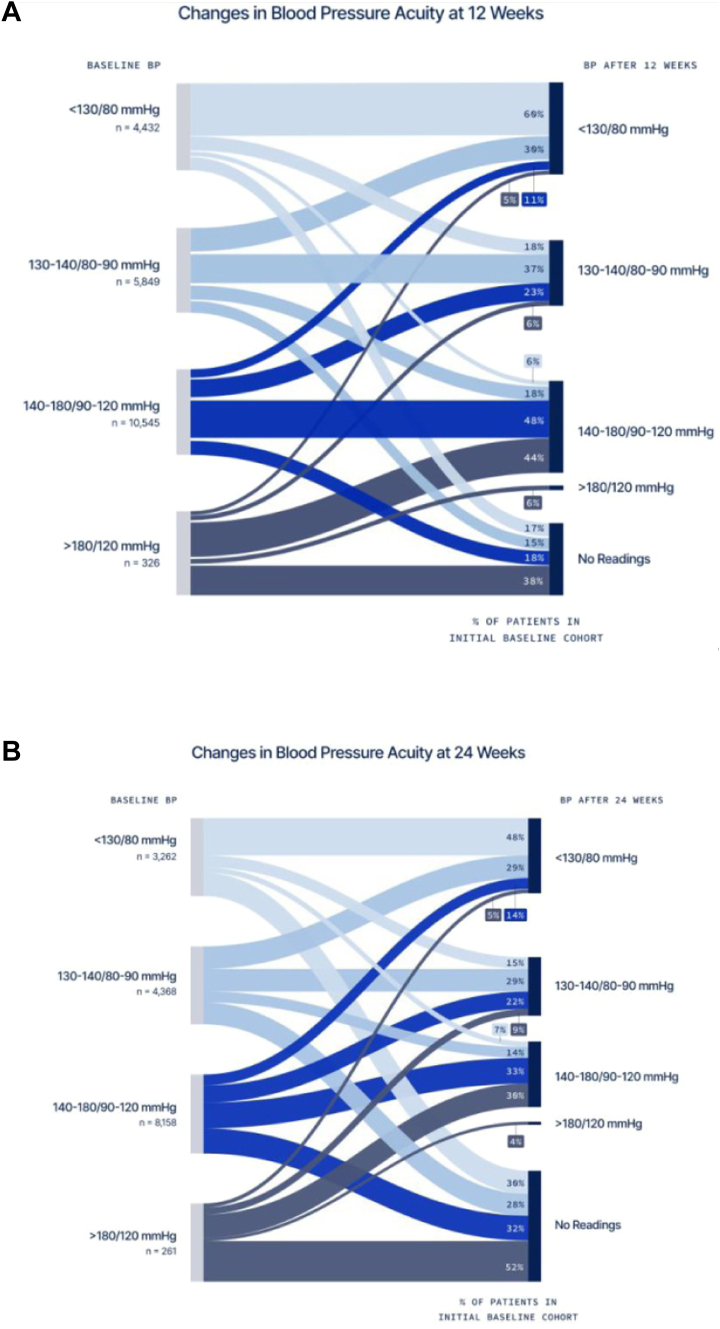
Changes in Mean Blood Pressure at 12 and 24 Weeks (A) Changes in baseline mean blood pressure at 12 weeks; (B) Changes in baseline mean blood pressure at 24 weeks. Abbreviation as in Figure 2.

The total mean (SD) reduction in SBP/DBP was 7/5 mm Hg (14/8) (*P* < 0.001) over a mean follow-up of 30 (24) weeks. There was a graded reduction in BP as the baseline increased with a 4/2 (11/6), 12/7 (14/8), and 29/14 (23/12) mm Hg decrease in SBP/DBP in individuals with a baseline BP of 130 to 140/80 to 90, 140 to 180/90 to 120, and >180/120 mm Hg, respectively. There was a 70% relative increase in the number of patients at BP goal (BP < 130/80 mm Hg) at follow-up compared to baseline (36.6% vs 21.5%; *P* < 0.001) (Figure 2A). The changes in baseline mean BP levels at 12 and 24 weeks are shown in Figure 3. Details regarding antihypertension medication changes are highlighted in Table 3.

**Table 3:** Medication Management

	Total Population (N = 23,638)	Baseline BP <130/80 mm Hg (n = 5,073)	Baseline BP 130-140/80-90 mm Hg (n = 6,574)	Baseline BP 140-180/90-120 mm Hg (n = 11,639)	Baseline BP >180/120 mm Hg (n = 352)
# of antihypertension medications prescribed, enrollment	43,475	8,650	11,509	22,606	710
# of antihypertension medications prescribed, follow-up	45,937	8,553	11,687	24,759	938
Total # of antihypertension medications up titrated	3,975	188	514	3,070	203
Total # of antihypertension medications down titrated	1,139	310	213	590	26
Total # of antihypertension medications initiated	2,539	112	282	2,012	133
Total # of antihypertension medications discontinued	1,674	312	313	992	57

### Blood pressure outcomes, stratified by baseline blood pressure

Based on home-based BP readings during the first 2 weeks in the program, 5,073 individuals (21.5%) were at goal at baseline. This same group had an in-clinic BP mean of 132/75 mm Hg. At follow-up, only 3,687 individuals still had a mean home-based BP at goal. Therefore, of the initial 21.5% of individuals at goal based on home BP measurements, ∼73% have elevated BP in office with normal BP at home. Rates of achieving goal BP according to baseline BP levels are displayed in Figure 2B.

### Blood pressure outcomes, stratified by geographic area

Among the 13,458 individuals from rural/underserved areas, the mean (SD) reduction in SBP/DBP was 7/4 mm Hg (15/8) (*P* < 0.001). The BP reduction was similar for individuals from nonrural/nonunderserved areas (7/4 mm Hg [14/8]). The percentage increase of patients achieving goal BP was similar among individuals from rural/underserved (71% increase; 36.5% vs 21.3%; *P* < 0.001) compared to nonrural/nonunderserved (71% increase; 36.9% vs 21.6%; *P* < 0.001) areas (rural/underserved vs nonrural/nonunderserved, *P* > 0.05) (Figure 2C).

### Blood pressure outcomes, stratified by program type

BP mean (SD) improvements were similar regardless of the program in which individuals were enrolled, with a 7/4 mm Hg (15/8) difference in the full and current medications-only programs and 6/4 mm Hg (14/8) difference in the recommend-only program. The greatest increase in the percentage who achieved BP goal was among individuals enrolled in the full medication program (79% increase; 36.6% vs 20.5%; *P* < 0.001) (Figure 2D).

### Program retention

Of the 23,638 patients enrolled in the program, 7,113 disengaged with 6,804 and 5,640 disengaging due to inactivity and/or disenrollment, respectively. Disengagement was most common among individuals with the highest baseline BP (n = 169, 48%), followed by those with baseline BP 140 to 180/90 to 120 mm Hg (n = 3,691, 32%), <130/80 mm Hg (n = 1,437, 28%), and 130 to 140/80 to 90 mm Hg (n = 1,816, 28%). Individuals disengaged after a mean of 207 days. Mean number of days until disengagement varied according to baseline BP, with those closest to achieving goal engaging for the longest time compared to those furthest from achieving goal engaging for the shortest time (Table 4).

**Table 4:** Program Retention

Rates of Disengagement^a^	n (%)	Mean Days (SD)
Total population	7,113 (30.1)	207 (155)
Baseline <130/80 mm Hg	1,437 (28.3)	203 (159)
Baseline 130-140/80-90 mm Hg	1,816 (27.6)	212 (159)
Baseline 140-180/90-120 mm Hg	3,691 (31.7)	208 (154)
Baseline >180/120 mm Hg	169 (48.0)	171 (144)
Disengagement–inactivity	6,804 (28.8)	224 (155)
Disengagement–disenrollment	5,640 (23.9)	213 (153)

a Disengagement included either inactivity or disenrollment. Inactivity was defined as not taking vitals within 30 days. Disenrollment was defined as actively disenrolling from the program. An individual patient can disengage either due to inactivity, disenrollment, or both inactivity and disenrollment.

## Discussion

Enrollment in a nationwide, comprehensive RPC hypertension program led by a multidisciplinary clinical team resulted in high levels of patient engagement and retention and improved BP control at scale. The positive effect on BP goals was most notable among individuals enrolled in the full medication management program. In addition, individuals living in rural/underserved geographic areas were able to achieve similar clinical outcomes compared to those in nonrural/nonunderserved communities despite having historically more limited access to health care.

In 2019, the Centers for Medicare and Medicaid Services initiated reimbursement for certain RPM codes tailored to individuals with chronic or acute conditions. While there has been growing acceptance of telehealth and virtual care by the clinical community and patients following the COVID-19 pandemic, there has also been an increase in the prevalence of hypertension and its associated costs, which further necessitate solutions that effectively address the gaps in clinical care.

One of the earliest studies evaluating the effect of RPM on hypertension control was completed in 2013 in a cluster randomized clinical trial where 450 adults across 16 clinics affiliated with a single health system in Minnesota were randomized to a telemonitoring intervention vs usual care.^16^ While the telemonitoring intervention achieved a higher percentage of patients at goal BP at 6 and 12 months (telemonitoring: 57.2% vs usual care: 30%), their BP goal was higher (<140/90 mm Hg) in comparison to this program as a result of evolving clinical recommendations over the last decade. In addition, although completed in a randomized fashion, the number of individuals in the analysis was significantly lower and all enrolled patients were from a single institution and state, which affects the generalizability when attempting to design a program that addresses the national burden of hypertension. Multiple investigators have since reported similar improvements in BP control using RPM and telehealth services,^17^, ^18^, ^19^ which resulted in endorsement of this service by the 2017 multisociety hypertension guideline.^1^

More recently, Massachusetts General Brigham (MGB) reported data on their RPM hypertension program, which included over 3,000 patients from varying racial, ethnic, and primary language backgrounds.^14^ Although >50% of individuals exited prematurely or became unreachable, in those who did continue in the program, they noted a significant reduction in BP (15.6/5.8 mm Hg) in individuals enrolled in the RPM compared to the education-only program after ∼3 months. They reported similar engagement and clinical outcomes regardless of race, ethnicity, and preferred language, which suggests a homogenous effect of the RPM program on hypertension control.

There are several important differences to note between the present RPC and MGB's RPM program. First, the MGB program, which reported similar engagement and clinical outcomes regardless of race, ethnicity, and preferred language, still had an overwhelming majority of patients who were non-Hispanic, White, and English speaking. In addition, all patients were enrolled in one state, from a similar geographic region and within a single large academic medical center, which limits the overall generalizability of the program to a representative nationwide population.

In contrast, the presently evaluated RPC program emphasized enrollment of individuals from rural/underserved areas across 21 states and multiple health systems. Today, individuals in rural/underserved areas have limited access to care and are most affected by SDOH, which leads to worse clinical outcomes and shorter life expectancy.^20^^,^^21^ This RPC program was capable not only of successfully enrolling individuals predominantly from rural/underserved areas but also of enabling patients in rural/underserved areas to achieve similar clinical outcomes and higher engagement and retention compared to their nonrural/nonunderserved counterparts. As a result, this RPC solution is more likely to result in improved hypertension control across a diverse population susceptible to SDOH and/or health disparities.

Second, compared to the MGB RPM program, this RPC program provided a solution at scale with over 6 times the number of patients enrolled while still achieving similar clinical outcomes and higher rates of clinical engagement and retention. Lastly, given the RPC partner-health system collaboration, this program provides a reproducible solution at scale that can aim to tackle the burden of chronic disease on a national level, instead of just within a single institution or state.

Despite multiple analyses demonstrating the clinical effectiveness of RPM for hypertension outcomes, one criticism of pursuing widespread RPM adoption has been inconsistent results related to variability in the clinical and support services provided. Although a comprehensive approach to care is required for effective RPM, identifying the right balance to optimize engagement, retention, and clinical outcomes is challenging.

In 2018, Mills et al^22^ performed a systematic review and meta-analysis to determine the effectiveness of different implementation strategies on BP control in hypertensive patients. They highlighted 8 patient-, provider-, or multilevel-implementation strategies, including team-based care, health coaching, electronic decision support systems, audit and feedback, which resulted in reductions in BP ranging from approximately 1 to 7 mm Hg. Without engagement data, however, it is difficult to ascertain whether different interventions or higher engagement in general leads to reductions in BP.

This program has corroborated that a multilevel strategy yields the greatest clinical benefit with regard to BP reduction and achievement of BP goal. When individuals with a baseline BP >180/120 mm Hg enroll in a comprehensive RPC program, they benefit from the largest and most significant reductions in BP, which are needed to achieve BP goals and avoid adverse clinical outcomes long-term. However, these patients who benefit most from an RPC program make up the smallest percentage of enrolled patients, once enrolled are more challenging to engage long-term, and when enrolled, are also more likely to be part of a more clinically restrictive arm of the RPC program where the clinical offering is less robust. Therefore, despite having the most at stake from a health perspective and achieving the greatest clinical benefit after enrollment, these patients are least likely to enroll and stay engaged. Notwithstanding decades of similar challenges in caring for and engaging with the sickest patients,^23^ the same problems still exist today. Only when there is optimal collaboration with patients, clinicians and health systems can RPC programs effectively target and treat those with the greatest clinical need and move the needle from a population health perspective.

On the other end of the spectrum are the ∼22% of enrolled patients who had a program baseline mean BP at goal even though their in-office BP was not at goal. A similar percentage of patients enrolled in the MGB RPM solution had hypertensive office readings with home BP readings at goal.^14^ This is referred to as white coat hypertension (WCH) and is more common among older individuals^24^ and can result in over medication and hypotension-related side effects. Furthermore, WCH has no association with adverse cardiovascular events.^25^ While this may be considered a low-risk subgroup to enroll in an RPC hypertension program, the data suggest that many of these patients benefit from regular at-home BP measurement and RPC as it often leads to clear documentation of WCH, which has a prevalence of up to 15% in the general population and 40% among a hypertensive population.^26^

In fact, patients enrolled at goal required down-titration or discontinuation of antihypertensive medications at follow-up as a result of frequent low BP readings at home. This, as well as variability of BP readings over time and different levels of program engagement, likely accounts for why patients who started the program at BP goal were later uncontrolled. Therefore, while additional investigation of this population is warranted, all patients with a diagnosis of hypertension should be considered for an RPC program to ensure the optimization of both vitals and symptoms and the avoidance of adverse side effects associated with overtreating patients with WCH.

### Study limitations

Several limitations of this comprehensive RPC hypertension program are worth highlighting. First, without a control group, the net effect of the program compared to the standard of care is difficult to delineate. It is important to note, however, that randomized controlled trials are unlikely to reach the appropriate scale needed to assess the effectiveness of RPC on hypertension management, making real-world implementation analysis like this more practical. The time, resources, and costs to execute a comprehensive RPC program at scale present challenges for health systems and academic medical centers where clinical and financial resources are already limited. As a result, the demand for RPC programs that depend on RPC partner-health system collaboration to support management of chronic diseases including hypertension is growing.

Second, although it is documented when patients seek emergency services, there are no safety and clinical outcomes data beyond BP management and control. That said, a reduction in BP is a well-documented surrogate for improved atherosclerotic cardiovascular disease outcomes. Third, given the various hypertension programs with different levels of clinical engagement and processes for titrating a medication, measurements of medication-associated BP changes are not uniform.

## Conclusions

Today, the standard of care leaves the U.S. adult population predisposed to significant preventable risk associated with uncontrolled hypertension. A fully remote, comprehensive RPC hypertension program may represent a scalable option for patients and providers to help significantly and safely achieve improved rates of BP control with the goal of improving cardiovascular outcomes. While these findings are similarly promising as compared to recent analyses studying the effects of RPM on hypertension control, the resources required to effectively scale a program like this at individual health systems are exceedingly high, which ultimately limits long-term clinical effectiveness. Working together with an RPC partner's team of dedicated clinicians, as modeled in this RPC program, can facilitate clinical impact at scale. This collaborative effort is key to shifting the national landscape of hypertension control, particularly for those affected by SDOH and health disparities.

Perspectives **COMPETENCY IN MEDICAL KNOWLEDGE:** Medicare patients across the United States have low rates of controlled hypertension. We demonstrate that a technology-enabled remote patient care program, which leverages remote patient monitoring and a proactive clinical care model, can drive high patient engagement and retention and improve clinical outcomes, specifically a greater percentage of patients achieving guideline-directed blood pressure goals. With further expansion and implementation, remote patient care has the ability to meaningfully impact hypertension treatment across the United States

**TRANSLATIONAL OUTLOOK:** Future studies should highlight not only the impact of remote patient care on clinical outcomes and engagement at scale but also the impact of remote patient care programs on cost of care for patients with hypertension.

## Funding support and author disclosures

Drs David I. Feldman, Simon, and Fudim are advisors at Cadence. Mr Reynolds, Mr Valor, and Drs Babikian, Theodore Feldman, Curnow, and Cunningham are employees at Cadence. All other authors have reported that they have no relationships relevant to the contents of this paper to disclose.
