# Supplementary material for: Clinical and Engagement Results of a Nationwide Comprehensive Remote Patient Care Hypertension Program
Source: JACC Adv. 2025 Jul 23;4(7):101892. doi: 10.1016/j.jacadv.2025.101892 (PMC12418473; doi:10.1016/j.jacadv.2025.101892)
Supplement: Supplementary data [file mmc1.docx]

**Supplemental Figure 1: Remote Patient Care Program Consort Diagram**


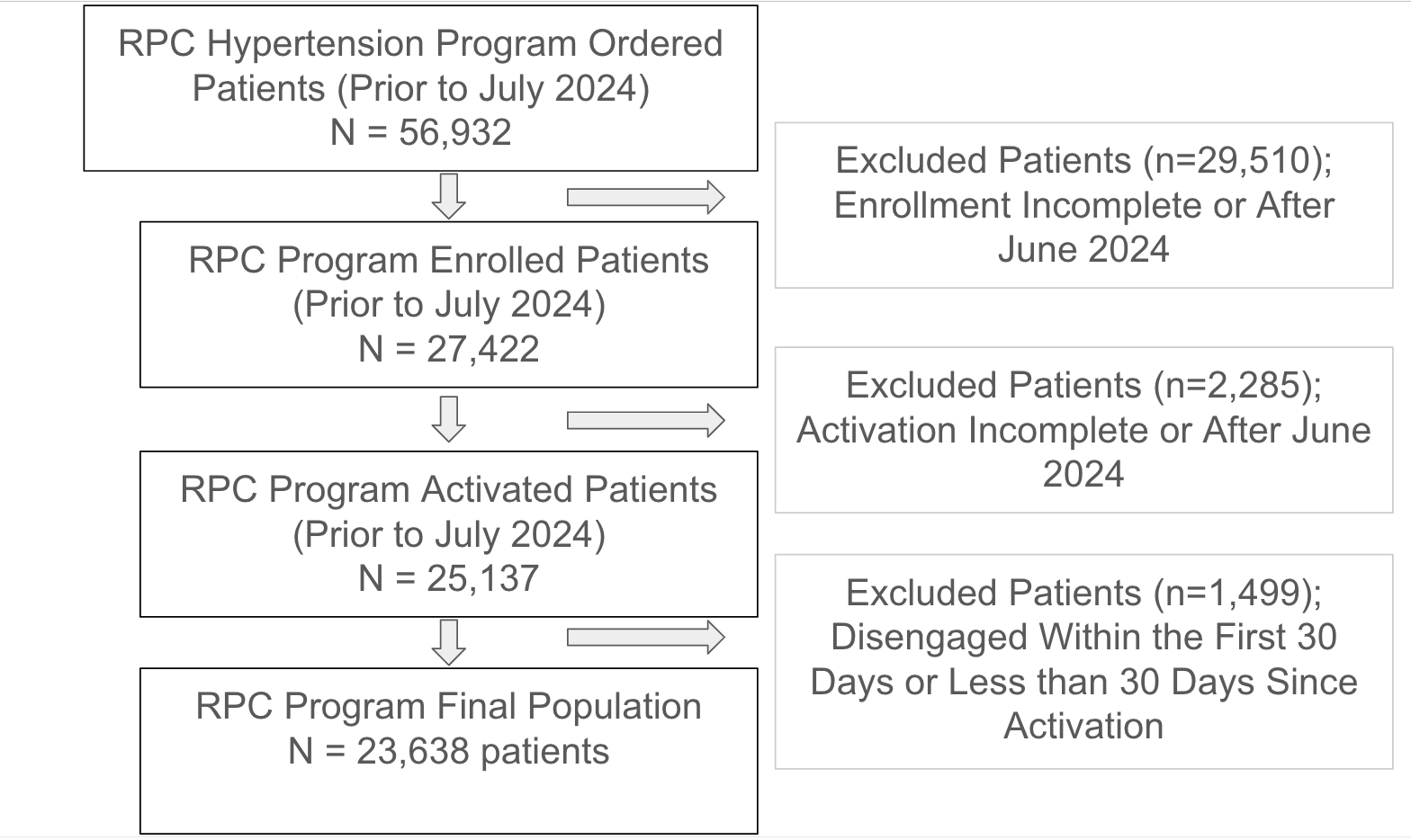


**Supplemental Figure 2: Remote Patient Care Hypertension Clinical Protocols and Workflow**

Panel A - This is an example of the clinical protocols for a given anti-hypertension medication that highlights the clinical considerations of the managing clinician as well as criteria utilized to determine eligibility of a medication titration. Panel B – This is the clinical workflow to guide clinician’s decisions around starting and/or titrating anti-hypertension medications.

**Panel A**

**Panel B**
